# A single-sEV analysis identifies plasma EPCAM^+^ sEVs as a biomarker for early diagnosis and monitoring postoperative remission of thyroid cancer

**DOI:** 10.20517/evcna.2025.93

**Published:** 2025-12-15

**Authors:** Simin Yu, Yuting Luo, Tianfeng Dang, Congli Peng, Qing Gan, Yuxuan Liang, Jieqing Yu, Ping Long, Wensheng Zhou, Daofeng Dai

**Affiliations:** ^1^Jiangxi Otorhinolaryngology-Head and Neck Surgery Institute, Department of Otorhinolaryngology-Head and Neck Surgery, The First Affiliated Hospital, Jiangxi Medical College, Nanchang University, Nanchang 330006, Jiangxi, China.; ^2^Department of General Thyroid Surgery, The First Affiliated Hospital, Jiangxi Medical College, Nanchang University, Nanchang 330006, Jiangxi, China.; ^#^Authors contributed equally to this work.

**Keywords:** Thyroid cancer, single sEV, biomarker, diagnosis, surveillance

## Abstract

**Aim:** Small extracellular vesicles (sEVs) are promising noninvasive biomarkers for several malignancies, including thyroid carcinoma (TC). However, their heterogeneity is frequently overlooked in bulk-level analyses.

**Methods:** Plasma samples from TC and healthy controls (HC) were collected for a proximity-dependent barcoding assay (PBA) to identify plasma sEV biomarkers at the single-sEV level. We screened the potential biomarkers using the Panel260 (the panel that detects 260 proteins) of PBA in Cohort 1, and validated them using Panel550 (the panel that detects 550 proteins) in Cohort 2.

**Results:** Plasma exosome counts were significantly elevated in TC compared with those in HC in both Cohort 1 and Cohort 2. Receiver operating characteristic curve analysis showed that sEV counts exhibited an area under the curve > 0.75 in both cohorts. The sEV proteomic analysis revealed that sEV epithelial cell adhesion molecule (EPCAM) levels were significantly increased, whereas claudin-11, integrin alpha X, and lymphocyte-activating 3 were significantly decreased in TC compared with HC. The increase in EPCAM in the plasma and tumor tissues was confirmed by enzyme-linked immunosorbent assay and immunohistochemistry analyses, respectively. The sEV subpopulation analysis further demonstrated that EPCAM^+^ sEVs were significantly elevated in TC compared with HC in both cohorts. The reduction in sEV counts was observed in 18 out of 20 patients after the operation. The decrease in EPCAM^+^ sEVs was observed in 20 patients with TC post-operatively, whereas the reduction in the conventional biomarker serum thyroglobulin (Tg) was observed in 14 patients. TC-derived plasma sEVs promoted TC cell proliferation, migration, invasion, and TC xenograft growth.

**Conclusion:** EPCAM^+^ sEVs could serve as a promising biomarker for the early diagnosis of TC and perform better in monitoring post-operative remission of TC than serum Tg.

## INTRODUCTION

Thyroid carcinoma (TC) is a prevalent endocrine malignancy with an estimated 821,000 new cancer cases and 44,000 deaths worldwide by 2022^[1]^. The main histological types of TC include papillary (PTC), follicular (FTC), medullary (MTC), Hurthle cell (HCTC), and anaplastic (ATC), which account for 80.2%, 11.4%, 3.5%, 3.1%, and 1.7% of all cases, respectively^[2]^. With the rapid increase in incidence of TC worldwide, biomarkers for its early diagnosis are urgently required. Serum thyroglobulin (Tg) is an established noninvasive biomarker for the detection of differentiated thyroid cancer (DTC) and disease surveillance. However, interference with Tg autoantibodies (TgAbs) limits their clinical utility^[3,4]^. TgAbs, which are present in up to 25% of patients with DTC, may underestimate or overestimate serum Tg concentrations. Thus, there is an urgent need to develop a novel noninvasive biomarker for the early detection of TC and surveillance of TC remission after surgery.

Small extracellular vesicles (sEVs) are extracellular vesicles (EVs) with diameters of 30-150 nm that play an important role in tumor cell proliferation, migration, and invasion due to their ability to transport cargo (such as nucleic acids, proteins, and lipids) from donor to recipient cells^[5,6]^. The sEVs are secreted by many cell types, including tumor and immune cells, and their cargo varies depending on the physiological status of the donor cells^[6]^, which renders sEVs as promising biomarkers for cancer diagnostics and surveillance. Ricklefs *et al.* reported that circulating EVs could function as biomarkers for the diagnosis and monitoring of patients with glioblastoma^[7]^. Li *et al.* found that EV miR-519e-5p was significantly elevated in distant metastatic PTC, promoted the malignant phenotype of PTC cells, and was transported to CD8^+^ T cells to aid tumor immune escape in distant organs^[8]^.

The sEVs possess heterogeneity in molecular composition owing to their different cell origins and stimuli to which the donor cells are subjected. Although the sEV contents (such as proteins, nucleic acids, and lipids) can be analyzed in bulk using high-throughput methods, information on sEV heterogeneity can be overlooked from bulk-level analyses. Recently, single-sEV analysis has become available using several approaches, such as asymmetric ﬂow ﬁeld-ﬂow fractionation^[9]^, ﬂuorescence-activated vesicle sorting by ﬂow cytometry^[10]^, acoustic microﬂuidics^[11]^, and nanoﬂow cytometry^[12]^. Recently, Wu *et al.* developed a proximity-dependent barcoding assay (PBA) that simultaneously analyzed more than 200 surface proteins of individual exosomes^[13]^. Guo *et al.* analyzed the EV surface proteins of patients with colorectal cancer (CRC) at single-EV resolution using PBA and found that integrin subunit beta 3 (ITGB3^+^) and integrin subunit alpha M (ITGAM^+^) EVs may be potential diagnostic, prognostic, and therapeutic biomarkers for CRC^[14]^. Cai *et al.* profiled EV surface proteins in five types of body fluids from patients with Alzheimer’s disease (AD) or AD model mice using PBA and found that EV proteins from urine samples possessed the highest diagnostic accuracy, reaching 88% with annexin A1 (ANXA1), integrin alpha X (ITGAX), and plasminogen activator, urinary (PLAU) in the protocadherin alpha 1 (PCDHA1^+^) subpopulation^[15]^. Ning *et al.* identified the tumor associated calcium signal transducer 2 (TACSTD2^+^) sEV subpopulation as a factor of tumor susceptibility in the elderly using PBA^[16]^. PBA was established to proﬁle the surface proteome of single urinary extracellular vesicles (uEVs), identifying CD35-uEV as a biomarker for sepsis-associated acute kidney injury^[17]^.

In this study, we collected plasma samples from patients with TC before surgery and compared their sEV surface protein profiles with those of healthy controls (HC) using PBA to identify a novel noninvasive biomarker for the early diagnosis of TC. Plasma samples from patients with TC after surgery were collected and the sEV surface protein profiles of patients with TC before and after surgery were analyzed to determine a new biomarker for monitoring post-operative remission of TC.

## METHODS

### Human samples

**Figure 1:**
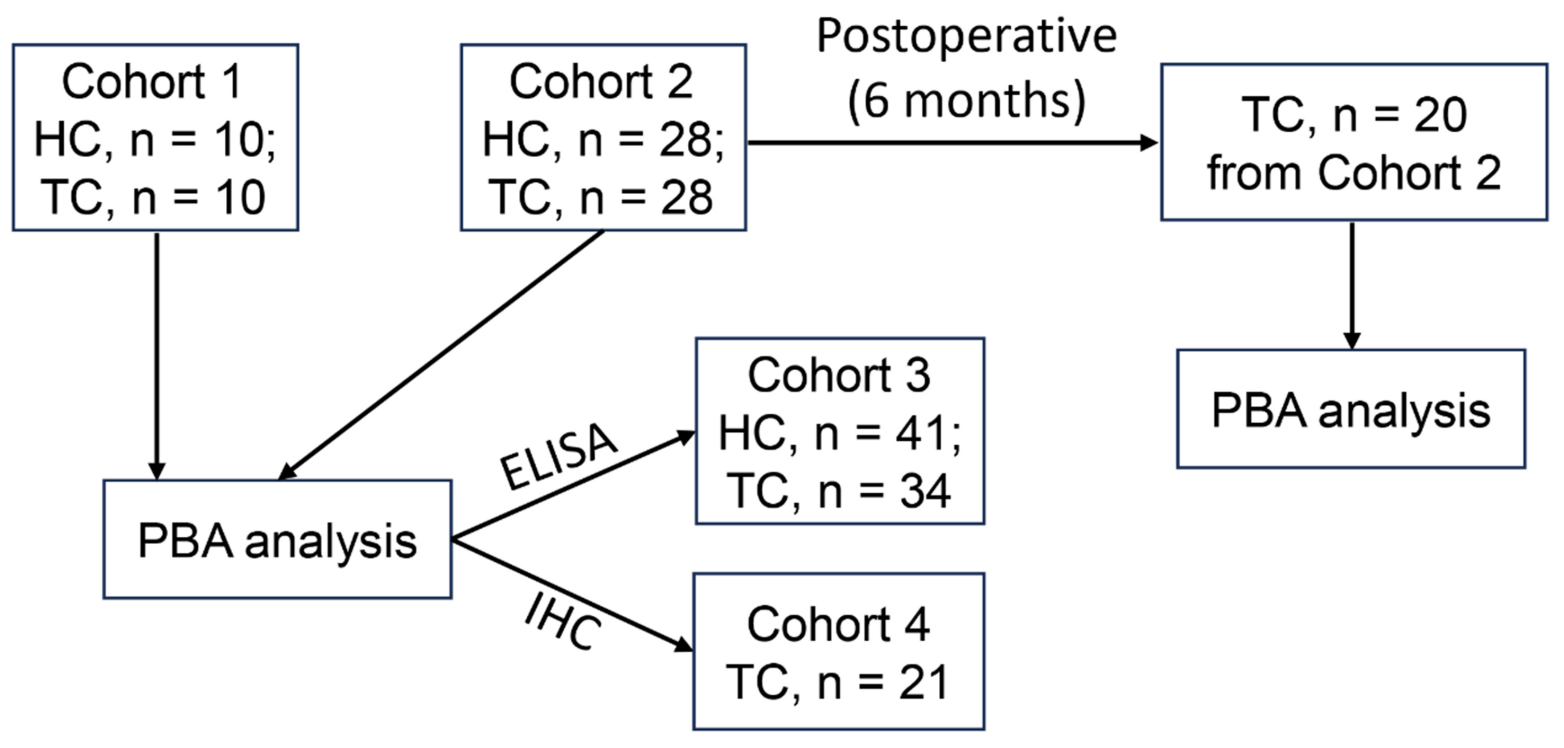
The flowchart explaining the recruitment of patients with TC for this study. TC: Thyroid carcinoma; HC: healthy controls; PBA: proximity-dependent barcoding assay; ELISA: enzyme-linked immunosorbent assay; IHC: immunohistochemistry.

Peripheral blood samples were collected from three cohorts. Cohort 1 comprised 10 HC and 10 patients with TC, while Cohort 2 included 28 HC and 28 TC. Plasma samples from Cohorts 1 and 2 were subjected to PBA analysis using Panel260 (the panel that detects 260 proteins) and Panel550 (the panel that detects 550 proteins), respectively. Cohort 3 consisted of 41 HC and 34 TC, and plasma samples from this cohort were subjected to enzyme-linked immunosorbent assay (ELISA) analysis. For PBA analysis, 38 patients contributed the preoperative blood samples, while 20 patients from Cohort 2 provided blood samples collected at month 6 post-surgery. Ninety-nine patients with TC enrolled in this study underwent thyroidectomy between December 2021 and June 2024 at the First Affiliated Hospital, Jiangxi Medical College, Nanchang University in Nanchang city, China. The inclusion criteria for patients with TC were as follows: 18 to 70 years of age and first diagnosis of TC. At the same time, 79 age- and sex-matched HCs were recruited. The exclusion criteria were as follows: known history of autoimmune thyroid disease; known history of other malignancy; receiving radiotherapy/chemotherapy before operation. Two patients with history of malignancy, and four patients receiving radiotherapy/chemotherapy before operation, were excluded. The archival slides of patients were analyzed by at least two pathologists. This study was approved by the Medical Research Ethics Committee of the First Affiliated Hospital, Jiangxi Medical College, Nanchang University [2022-3-036 and (2024)CDYFYYLK(06-013)] as per the Declaration of Helsinki. All patients signed informed written consent before recruitment. Plasma samples were prepared by drawing blood into the collection tubes containing dipotassium ethylenediaminetetraacetate (EDTA-K2) as an anticoagulant. Subsequently, the tubes were centrifuged at 3,000 *g* for 10 min. The supernatant was stored at -80 °C for further analysis. We also utilized formalin-fixed paraffin-embedded (FFPE) specimens of 21 TC patients (Cohort 4) from the Pathology Department for immunohistochemistry (IHC) of epithelial cell adhesion molecule (EPCAM) expression. The clinical features of TC patients enrolled in this study were listed in Supplementary Table 1. Figure 1 showed the enrollment of patients with TC for this study.

### PBA and sEV proteome data analysis

The surface proteins on individual sEVs were assessed using PBA technology (Secretech, China). Briefly, the procedure was as follows: The PBA probes included a panel of 260 or 550 antibodies conjugated with oligonucleotides containing unique molecular tags, unique protein tags, and also universal primers. Plasma was incubated with PBA probes for 2 h. To prepare exosome capture plate, biotinylated cholera toxin subunit B [biotin-CTB, 2.5 μg/mL in phosphate-buffered saline (PBS), C34779, Thermo, USA] was added to each well of a 96-well plate with streptavidin coating (PCR0STF-SA5/100, Biomat, Italy). The plate was incubated for 20 min at room temperature and washed three times with PBS with Tween 20 (PBST). Plasma/probe mix was diluted to a volume of 20 μL with PBA buffer and transferred into each well of cholera toxin subunit B (CTB)-coated plate for affinity capture of exosomes through the interaction between CTB and monosialotetrahexosylgangliosides (GM1 gangliosides) enriched in the lipid membrane of exosomes^[14]^. Rolling circle amplification (RCA) products, which contained the single sEV barcode, were added to the plate and annealed to the oligonucleotides in PBA probes. The oligonucleotides in PBA probes were extended using the RCA products as the template. The generated oligonucleotides were utilized to construct the libraries which underwent PE150 sequencing on the NovaSeq S4 platform (Illumina, USA). Subsequently, the bcl2fastq software (version 2.20.0.42, Illumina, USA) was employed to convert the raw sequencing data in bcl files into fastq files. In this study, we used the EVisualizer® decoding package (version 1.0, Secretech, China) to analyze sequencing reads and construct the sEV identity-protein expression dataset, which forms the basis for the PBA method analysis. Initially, we processed the raw data using the EVisualizer® algorithm and performed statistical analysis to summarize the number of detected sEVs and proteins as mean ± standard error (SE). We then integrated the total expression levels of each protein into a protein expression dataset. This dataset was normalized using the trimmed mean of M-values (M denotes the log ratio between samples) algorithm to obtain a standardized total protein expression dataset. The sEVs with a particular protein were defined as sEV subpopulations, forming the sEV subpopulation dataset. This dataset underwent counts per million (CPM) normalization. CPM value = 10^6^ × (The number of target sEVs)/(The number of total sEVs). The sEV subpopulations were also generated using the unsupervised FlowSOM algorithm, a computational algorithm used for the unsupervised clustering and visualization of high-dimensional flow cytometry data^[18]^. The t-distributed stochastic neighbor embedding (t-SNE) method^[19]^ was employed for the plotting of sEV subpopulations.

### Cell culture and transfection

Human TC cell lines TPC-1 and BCPAP were purchased from Procell Life Science & Technology Co., Ltd. and Fuheng Biology, respectively, and they were authenticated using short tandem repeat (STR) method before experiment. TPC-1 cells were cultured in Roswell Park Memorial Institute 1640 (RPMI-1640) medium, while BCPAP cells were cultured in Dulbecco’s Modified Eagle Medium (DMEM) medium, supplemented with 10% fetal bovine serum (FBS) and 1% penicillin-streptomycin (PS). The above cells were cultured at 37 °C in a 5% CO_2_ chamber. The regular FBS was ultracentrifuged at 100,000 × *g* for 18 h at 4 °C to remove sEVs, generating sEV-depleted FBS, which was used for culturing TC cells^[20]^. The 293T cells were infected with lentivirus of negative control short hairpin RNA (shNC) or lentivirus of EPCAM short hairpin RNA (shEPCAM) obtained from OriGene Technologies (USA), and then screened by puromycin. The knockdown of EPCAM was confirmed using western blotting (anti-EPCAM, #21050-1-AP, Proteintech, China; anti-Beta-Actin, #66009-1-Ig, Proteintech, China).

### Purification and characterization of sEVs

The sEVs were collected using a differential centrifugation method. The samples were first centrifuged at 300 × *g* for 10 min, then at 2,000 × *g* for 10 min, and finally at 10,000 × *g* for 30 min to remove cells or cell debris. Subsequently, the supernatant was subjected to centrifugation at 100,000 × *g* for 70 min using an Optima XPN-100 ultracentrifuge equipped with a 32Ti rotor (Beckman Coulter, Germany) to obtain the sEVs which were resuspended with PBS. Repeat the ultracentrifugation step. Finally, all purified sEVs were resuspended in PBS. The presence of the proteins cluster of differentiation 9 (CD9, #380441, ZEN BIO, China), tumor susceptibility gene 101 (TSG101, #R25999, ZEN BIO, China), Golgi Matrix 130 (GM130, #HA721282, HuaBio, China), and Calnexin (#10427-2-AP, Proteintech, China) in the isolated sEVs was analyzed by western blot analysis. The nanoparticle tracking analysis (NTA) of sEVs was performed using ZETASIZER Nano ZS (Malvern, UK) to obtain sEV particle concentrations and their size distribution. The microphotographs of sEVs were obtained using an HT7700 transmission electron microscope (Hitachi, Japan).

### Cell uptake experiment

The sEVs were labeled with 1:100 dilution of 3,3′-dioctadecyloxacarbocyanine perchlorate (Dio, Beyotime, China). The labeled sEVs were washed twice with RPMI-1640 medium to remove excess Dio, resuspended in PBS, and then co-cultured with TPC-1 or BCPAP cells at 37 °C for 24 h. After fixation with 4% paraformaldehyde, the cells were stained with 4′,6-diamidino-2-phenylindole (DAPI) nuclear staining dye (Beyotime, China). Following PBS washing, cell uptake was observed and images were captured using an inverted fluorescence microscope (Carl Zeiss, Germany).

### Transwell assays

TC cells were suspended in the medium containing 0.5% FBS and 4.0 × 10^4^ cells were seeded onto each transwell insert (Corning, USA) with pore size of 8 μm, establishing the upper chamber. The lower chamber contained 600 μL medium with 20% FBS. After incubating the cells for 12 h, they were fixed with 4% paraformaldehyde at room temperature for 30 min, and stained with 0.1% crystal violet for 20 min. The transwell chambers were washed, air-dried, inverted, and photographed under a microscope at a magnification of 200×. Cell counting was performed using ImageJ. For the invasion assay, Matrigel (Yeasen, China) and serum-free medium were mixed at a ratio of 1:2 and 50 μL of the mixture was thinly coated on the bottom of the upper chamber. After the Matrigel solidified, 8.0 × 10^4^ cells were added to the upper chamber. The remaining steps were the same as in the migration assay, except that cells were cultured for 24 h at 37 °C.

### Cell proliferation

Cell proliferation was assessed using the cell counting kit-8 (CCK-8) kit (Abmole, USA). A total of 3.0 × 10^3^ cells were seeded into each well of a 96-well plate, with 4 replicate wells per group and 100 µL of medium per well. Cells were incubated in a cell culture incubator (37 °C, 5% CO_2_). Then, 10 µL of CCK-8 solution was added to each well. After cells were incubated in the incubator (37 °C, 5% CO_2_) for 1 h, the absorbance at 450 nm was measured using a microplate reader. Additionally, colony formation assays were performed to assess cell proliferation. Cells in log phase were collected and seeded into 6-well plates at a density of 500 cells per well. The medium was changed every 3 days, and cell status was observed. When visible colonies appeared, the culturing was terminated. Colonies were fixed with 4% paraformaldehyde for 30 min, stained with 0.1% crystal violet for 20 min, and counted under a microscope. The number of colonies was counted using ImageJ software.

### ELISA

The levels of EPCAM in plasma samples were measured using the Human EPCAM ELISA Kit (Meike, China) according to the manufacturer’s instructions. The optical density at 450 nm (OD_450_) was measured using a microplate reader (Thermo Fisher, USA).

### IHC

IHC analysis was performed using the following antibodies: anti-EPCAM (1:200, #R24218, ZEN BIO, China). Subsequently, the slides were photographed under a microscope (Carl Zeiss, Germany) and analyzed using ImageJ software (National Institutes of Health, Bethesda, MD, USA) and Image-ProPlus software (Media Cybernetics, Rockville, MD, USA).

### Animal experiments

The male Bagg Albino/c (BALB/c) nude mice (4-6 weeks old) were obtained from GemPharmatech (Jiangsu, China) and were raised under pathogen-free conditions. All animal experimental protocols were approved by the Animal Ethics Committee of the First Affiliated Hospital of Nanchang University (CDYFY-IACUC-202311QR035). Twelve BALB/c nude mice were randomly divided into two groups using the random number table method: the PBS control group and the sEV injection group. TPC-1 cells (5.0 × 10^6^) were subcutaneously injected into the left axilla of BALB/c nude mice. PBS or plasma sEVs derived from TC patients were injected into the mice via the tail vein from day 1 to the day when the mice were sacrificed. According to the work by Guo *et al.*, the sEVs were injected every other day, with each injection containing 50 μg of sEVs dissolved in 100 µL PBS^[14]^. The mice were euthanized at day 32. Tumor volume was measured every two days.

### Statistical analysis

Student’s *t*-test was used for comparing differences between two groups with a normal distribution, and the Mann–Whitney *U* test was used for comparing differences between two groups with a non-normal distribution. The normality test was performed using the Shapiro–Wilk test. The Benjamini-Hochberg (BH) method was used to adjust the *P*-values.

## RESULTS

### Comparison of the number of plasma sEVs between HC and TC

**Figure 2:**
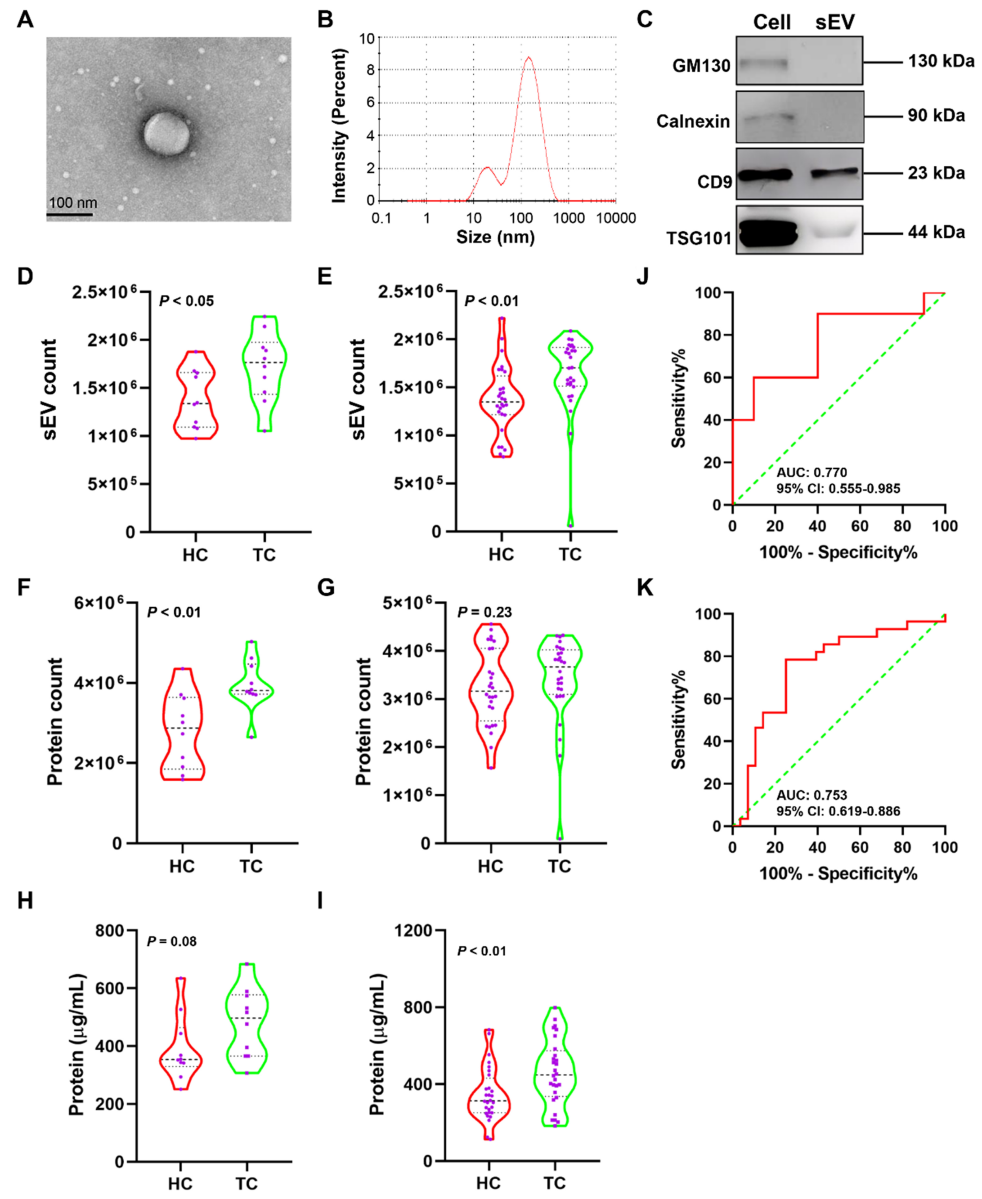
Analysis of the plasma sEV count at single-sEV level using PBA. (A) TEM of plasma sEVs. Solid black lines represent 100 nm scale bars; (B) Size distribution of plasma sEVs by NTA; (C) Western blot analysis of plasma sEVs using antibodies CD9, TSG101, GM130, and Calnexin. Plasma samples from Cohorts 1 and 2 were subjected to PBA analysis using Panel260 and Panel550, respectively; Comparison of plasma sEV count detected by PBA between HC and patients with TC in Cohort 1 (D) and 2 (E), the data of plasma sEV count in Cohort 1 was analyzed using the Student’s *t*-test while the data in Cohort 2 was analyzed using the Mann‒Whitney *U* test; The differences in the number of sEV surface proteins detected by PBA between HC and TC in Cohort 1 (F) and 2 (G), the data of protein count in Cohort 1 was analyzed using the Student’s *t*-test while the data in Cohort 2 was analyzed using the Mann‒Whitney *U* test; The protein concentration of isolated sEVs was detected using the BCA kit, and was compared between HC and TC in Cohort 1 (H) and 2 (I), the data of protein concentration in Cohort 1 and 2 was analyzed using the Student’s *t*-test; ROC curves were plotted using the detected plasma sEV counts of HC and TC in Cohort 1 (J) and 2 (K). sEV: Small extracellular vesicle; PBA: proximity-dependent barcoding assay; TEM: transmission electron microscopy; NTA: nanoparticle tracking analysis; CD9: cluster of differentiation 9; TSG101: tumor susceptibility gene 101; GM130: Golgi Matrix 130; HC: healthy controls; TC: thyroid carcinoma; BCA: Bicinchoninic acid assay; ROC: receiver operating characteristic; AUC: area under the curve; CI: confidence interval.

The properties of plasma sEVs from HC were characterized using transmission electron microscopy (TEM), NTA, and western blotting [Figure 2A-C]. The image of plasma sEV was obtained using TEM [Figure 2A]. NTA showed that sEVs from the plasma samples had an average diameter of 78.6 nm [Figure 2B]. Furthermore, two sEV markers, CD9 and TSG101, were present in isolated sEVs, and the negative control marker, GM130 and Calnexin, was detected in TPC-1 cells but not in plasma sEVs [Figure 2C]. Plasma samples from Cohorts 1 and 2 were subjected to PBA analysis using Panel260 and Panel550, respectively, to determine sEV surface proteins. Firstly, we analyzed sEV counts obtained by PBA which uses complex Tags to identify individual exosomes and count sEVs^[13]^. Patients with TC in Cohort 1 had a significantly higher median number of plasma sEVs than HC (1.77 × 10^6^ *vs.* 1.34 × 10^6^; *P* < 0.05; Figure 2D); a similar trend was observed in Cohort 2 (1.65 × 10^6^ *vs.* 1.38 × 10^6^; *P* < 0.01; Figure 2E). The median number of sEV surface proteins was significantly elevated in patients with TC compared with that in HC in Cohort 1 (3.81 × 10^6^ *vs.* 2.88 × 10^6^; *P* < 0.01; Figure 2F). In Cohort 2, a higher number of median sEV surface proteins was observed in patients with TC than in the HC group; however, the difference was not significant (3.51 × 10^6^ *vs.* 3.17 × 10^6^; *P* = 0.28; Figure 2G). We also detected protein concentration of isolated sEVs using the Bicinchoninic acid assay (BCA) kit. Figure 2H showed that protein concentration was higher in TC than in HC in Cohort 1; Nevertheless, the difference was not significant (*P* = 0.08). In cohort 2, the protein concentration was significantly higher in TC than in HC (*P* < 0.01; Figure 2I). Receiver operating characteristic (ROC) curve analysis was performed to evaluate the diagnostic value of sEV number in distinguishing TC from HC. The detected sEVs showed an area under the curve (AUC) > 0.750 in both cohorts [Figure 2J and K].

### Identifying sEV surface protein profiles of patients with TC at single-sEV level using PBA

**Figure 3:**
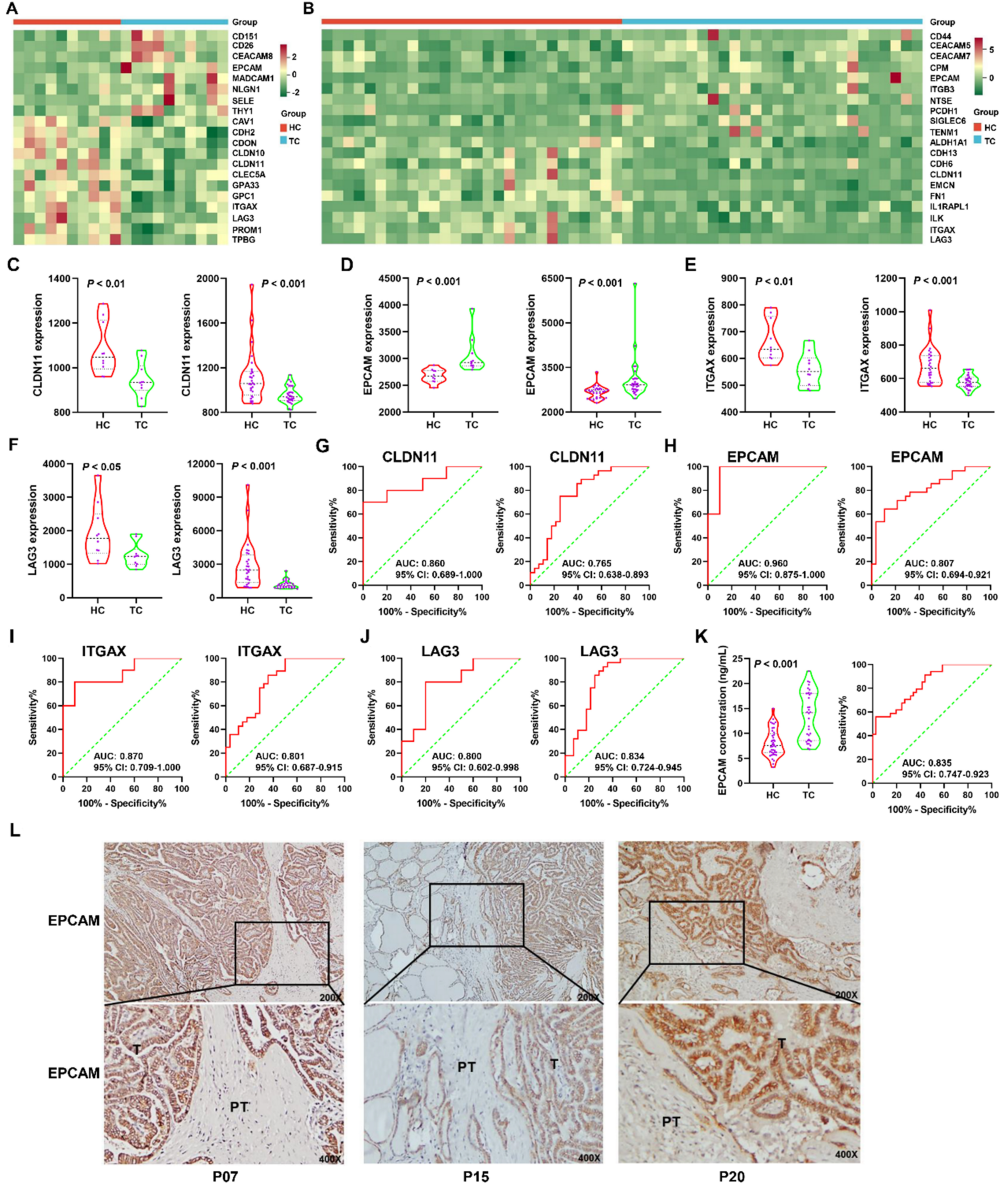
Characterization of sEV surface protein biomarkers for early diagnosis of TC via PBA. The heatmaps displayed the top 20 differentially expressed sEV proteins between HC and TC in Cohort 1 (A) and 2 (B); The differential expression of CLDN11 (C), EPCAM (D), ITGAX (E), and LAG3 (F) between HC and TC in Cohort 1 (left panel) and 2 (right panel). The data of CLDN11, ITGAX, and LAG3 in Cohort 1 was analyzed using the Student’s *t*-test while their data in Cohort 2 was analyzed using the Mann‒Whitney *U* test. The data of EPCAM in both cohorts was analyzed using the Mann‒Whitney *U* test; ROC curves for CLDN11 (G), EPCAM (H), ITGAX (I), and LAG3 (J) in Cohort 1 (left panel) and 2 (right panel) were plotted; (K) The plasma EPCAM concentrations were analyzed using an ELISA kit in Cohort 3, which included 41 HC and 34 TC patients. The data of plasma EPCAM concentration was analyzed using the Mann‒Whitney *U* test. The ROC curve was plotted in the right panel; (L) EPCAM expression was assessed by IHC in tissue sections of TC (T) with peritumor tissues (PT) and representative images were exhibited for samples in Cohort 4 (*n* = 21). sEV: Small extracellular vesicle; TC: thyroid carcinoma; PBA: proximity-dependent barcoding assay; HC: healthy controls; CLDN11: claudin 11; EPCAM: epithelial cell adhesion molecule; ITGAX: integrin alpha X; LAG3: lymphocyte-activating 3; ROC: receiver operating characteristic; ELISA: enzyme-linked immunosorbent assay; IHC: immunohistochemistry; AUC: area under the curve; CI: confidence interval.

The heat maps in Figure 3A and B show the top 20 significantly differentially expressed sEV surface proteins in Cohorts 1 and 2, respectively [Supplementary Tables 2 and 3]. Four proteins, claudin 11 (CLDN11, UniProt ID: O75508), EPCAM (UniProt ID: P16422), ITGAX (UniProt ID: P20702), and lymphocyte-activating 3 (LAG3, UniProt ID: P18627), co-occurred in both heat maps, indicating that these proteins were significantly differentially expressed in both cohorts. CLDN11 belongs to a family of claudins that are highly expressed in some cancers^[21]^ and play an important role in tight junction-initiated epithelial-mesenchymal transition (EMT), which mediates tumor cell migration^[22]^. The sEV CLDN11 level was significantly lower in patients with TC than in HC in both Cohorts 1 and 2 (*P* < 0.01; Figure 3C). A significant elevation in EPCAM expression in TC was observed in both cohorts (*P* < 0.001; Figure 3D). EPCAM is a transmembrane glycoprotein mainly expressed in epithelia and epithelial-derived tumors, including CRC, lung carcinoma, and other epithelial tumors^[23]^. ITGAX (also known as CD11c), a biomarker of dendritic cells (DCs), significantly decreased in TC compared to that in HC in both cohorts (*P* < 0.01; Figure 3E). A significant decrease in LAG3 expression in TC was also noted in both cohorts (*P* < 0.05; Figure 3F). LAG3, also known as CD223, is a potential cancer immunotherapeutic target because of its ability to mediate T-cell exhaustion^[24]^. ROC curve analysis showed that the AUCs for CLDN11 in Cohorts 1 and 2 were 0.860 [95% confidence interval (CI): 0.689-1.000; Figure 3G, left panel] and 0.765 (95%CI: 0.638-0.893; Figure 3G, right panel), respectively. EPCAM, ITGAX, and LAG3 had AUCs > 0.800 in both cohorts [Figure 3H-J]. The AUCs for EPCAM in Cohorts 1 and 2 were 0.960 (95%CI: 0.875-1.000; Figure 3H, left panel) and 0.807 (95%CI: 0.694-0.921; Figure 3H, right panel), respectively. The AUCs for ITGAX in Cohorts 1 and 2 were 0.870 (95%CI: 0.709-1.000; Figure 3I, left panel) and 0.801 (95%CI: 0.687-0.915; Figure 3I, right panel), respectively. The AUCs for LAG3 in Cohorts 1 and 2 were 0.800 (95%CI: 0.602-0.998; Figure 3J, left panel) and 0.834 (95%CI: 0.724-0.945; Figure 3J, right panel), respectively. ELISA analysis in Cohort 3 validated that plasma EPCAM was significantly higher in TC than in HC [Figure 3K, left panel], and ROC curve analysis showed that the AUC for EPCAM in Cohort 3 was 0.835 (95%CI: 0.747-0.923; Figure 3K, right panel). IHC analysis in Cohort 4 showed that EPCAM expression was higher in tumor tissues than in peritumoral tissues [Figure 3L].

### Identification of the sEV subpopulation biomarkers for TC

**Figure 4:**
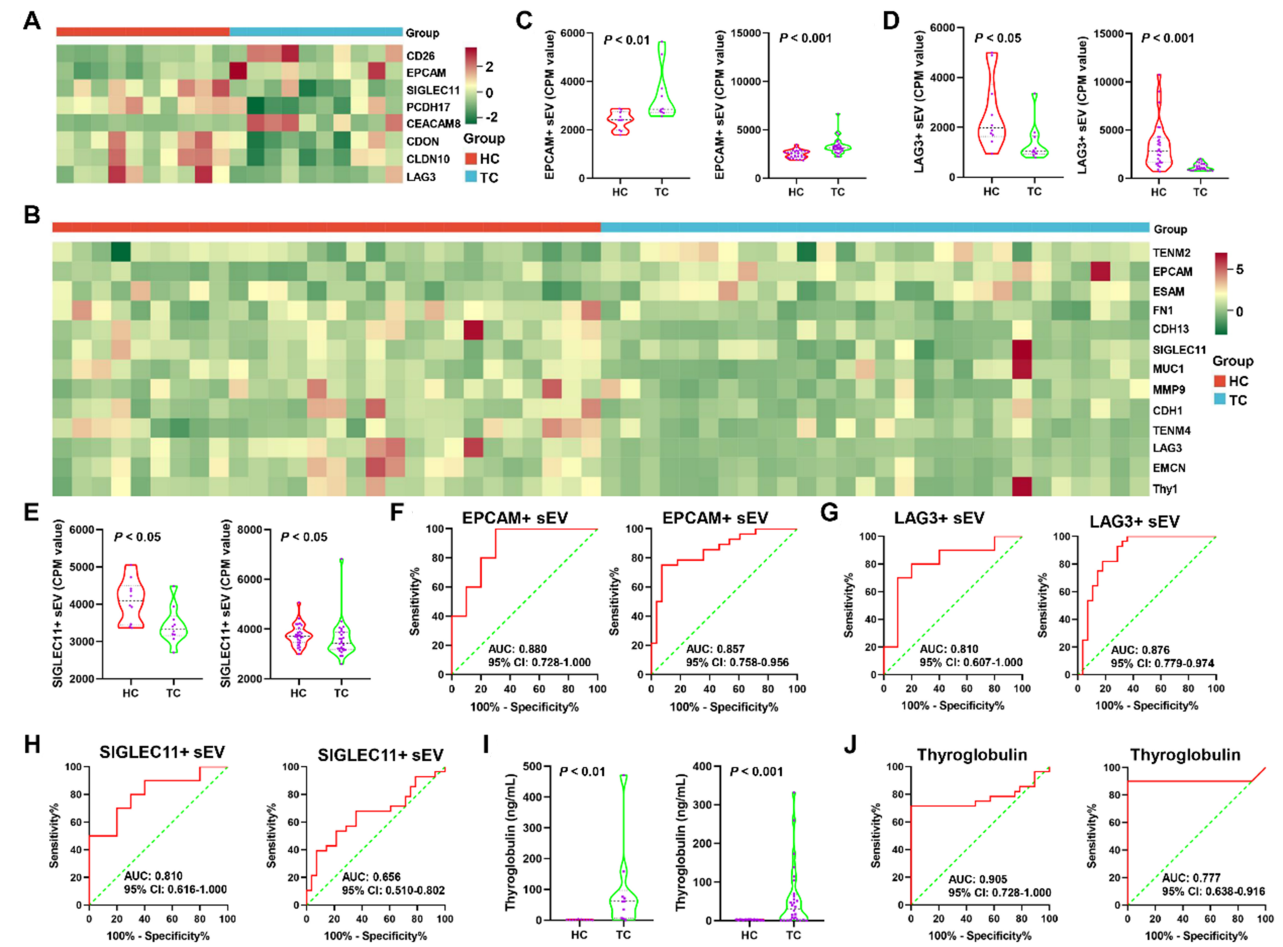
Identification of plasma sEV subpopulation biomarkers for early diagnosis of TC. The differential plasma sEV subpopulations between HC and TC in Cohort 1 (A) and 2 (B) were displayed in the two heatmaps; The CPM values of EPCAM^+^ (C), and LAG3^+^ (D), SIGLEC11^+^ (E) sEVs were compared between HC and TC in Cohort 1 (left panel) and 2 (right panel). The data of EPCAM^+^ and LAG3^+^ sEVs in both cohorts was analyzed using the Mann‒Whitney *U* test. The data of SIGLEC11^+^ sEVs in cohort 1 and 2 was analyzed using the Student’s *t*-test and the Mann‒Whitney *U* test, respectively; ROC curves for EPCAM^+^ (F), and LAG3^+^ (G), SIGLEC11^+^ (H) sEVs in Cohort 1 (left panel) and 2 (right panel) were plotted; (I) The conventional biomarker, serum Tg, was compared between HC and TC in Cohort 1 (left panel) and 2 (right panel). The data of serum Tg in both cohorts was analyzed using the Mann‒Whitney *U* test; (J) ROC analysis of Tg in Cohort 1 (left panel) and 2 (right panel). sEV: Small extracellular vesicle; TC: thyroid carcinoma; HC: healthy controls; CPM: counts per million; EPCAM: epithelial cell adhesion molecule; LAG3: lymphocyte-activating 3; SIGLEC11: sialic acid-binding Ig-like lectin 11; ROC: receiver operating characteristic; Tg: thyroglobulin; AUC: area under the curve; CI: confidence interval.

**Figure 5:**
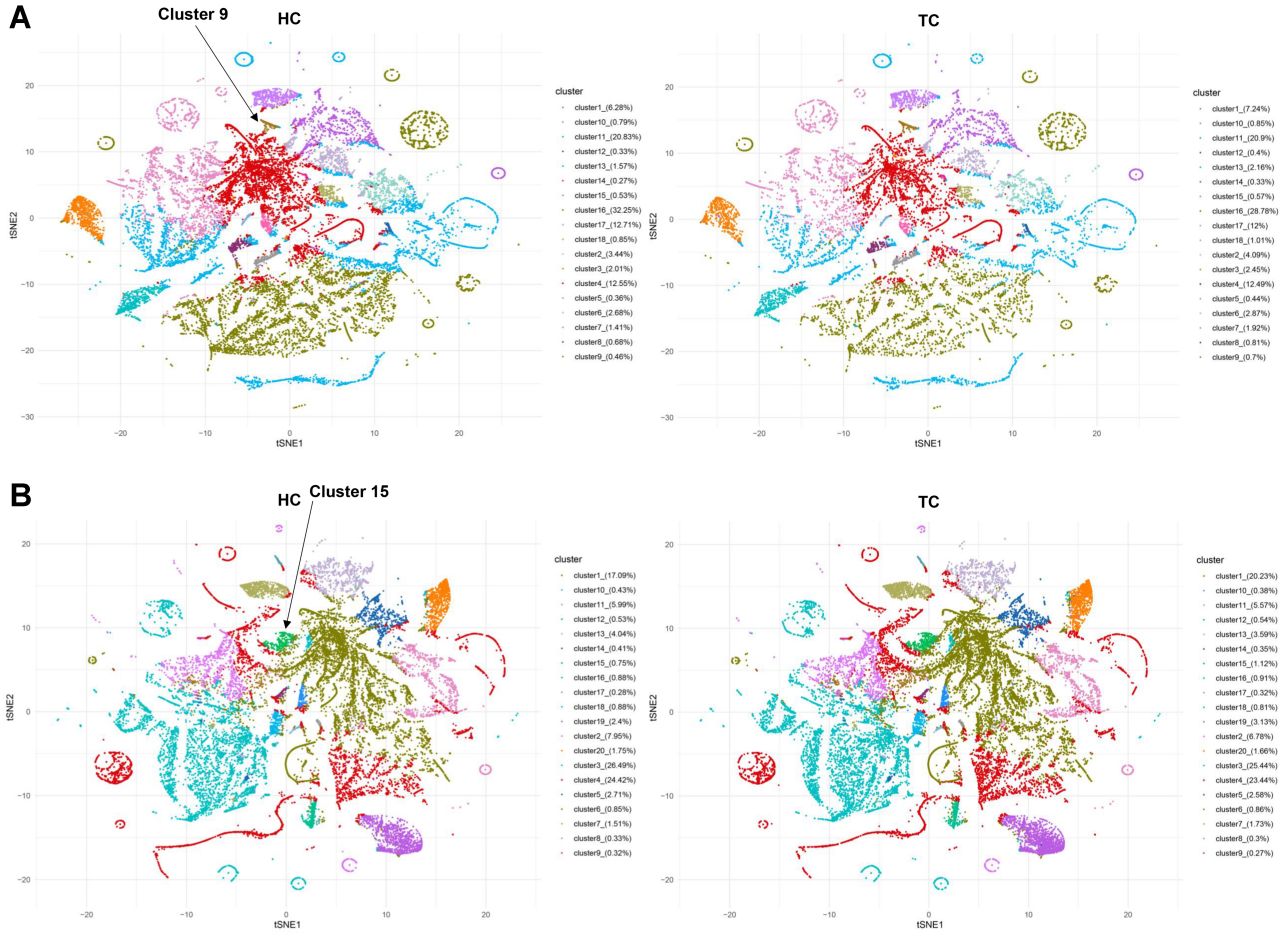
Differences in distribution and population of sEV clusters between HC and TC. The sEV clusters for Cohort 1 (A) and 2 (B) were generated using FlowSOM algorithm. sEV: Small extracellular vesicle; HC: healthy controls; TC: thyroid carcinoma.

To identify the sEV subpopulation biomarkers for TC, we analyzed sEVs with the identified proteins in our gene panel and quantified them using the CPM value. The heat maps in Figure 4A and B show significantly different sEV subpopulations between TC and HC in Cohorts 1 and 2, respectively [Supplementary Tables 4 and 5]. The sEVs with EPCAM (EPCAM^+^ sEVs), sialic acid-binding Ig-like lectin 11 (SIGLEC11^+^ sEVs), and LAG3 (LAG3^+^ sEVs) were observed in both heat maps, suggesting that EPCAM^+^, SIGLEC11^+^, and LAG3^+^ sEVs were notably differential subpopulations in the two cohorts. EPCAM^+^ sEVs were significantly elevated in both cohorts (*P* < 0.01; Figure 4C). A significant decrease in LAG3^+^ and SIGLEC11^+^ sEVs was observed in both cohorts (*P* < 0.05; Figure 4D and E). EPCAM^+^ and LAG3^+^ sEVs had AUCs > 0.800 in both cohorts [Figure 4F and G]. The AUCs for SIGLEC11^+^ sEVs in Cohorts 1 and 2 were 0.810 (95%CI: 0.616-1.000; Figure 4H, left panel) and 0.656 (95%CI: 0.510-0.802; Figure 4H, right panel), respectively. We also evaluated the performance of the serum Tg, which is a conventional marker for distinguishing TC from HC. Figure 4I showed that the level of serum Tg was significantly higher in TC than in HC in both cohorts (*P* < 0.01). The AUCs for Tg in Cohorts 1 and 2 were 0.905 (95%CI: 0.728-1.000; specificity = 90.00% and sensitivity = 90.00%; Figure 4J, left panel) and 0.777 (95%CI: 0.638-0.916; specificity = 71.43% and sensitivity = 85.71%; Figure 4J, right panel), respectively. EPCAM^+^ sEVs possessed a specificity of 80.00% and a sensitivity of 80.00% in Cohort 1 and a specificity of 78.57% and a sensitivity of 82.14% in Cohort 2 [Figure 4F]. The sEV subpopulations were also analyzed using the FlowSOM algorithm, which generated 18 and 20 sEV clusters for Cohorts 1 and 2, respectively [Figure 5, Supplementary Tables 6 and 7]. In Cohort 1, the proportions of cluster 9, in which EPCAM were dominantly expressed, were higher in TC than in HC (0.46% *vs.* 0.7%; Figure 5A). In Cohort 2, EPCAMs were dominantly expressed in cluster 15, and the proportions of cluster 15 were higher in TC than in HC (0.75% *vs.* 1.12%; Figure 5B). These results indicate that EPCAM^+^ sEVs may serve as a promising biomarker for the early diagnosis of TC.

### Characterization of sEV biomarkers to monitor post-operative remission of TC

**Figure 6:**
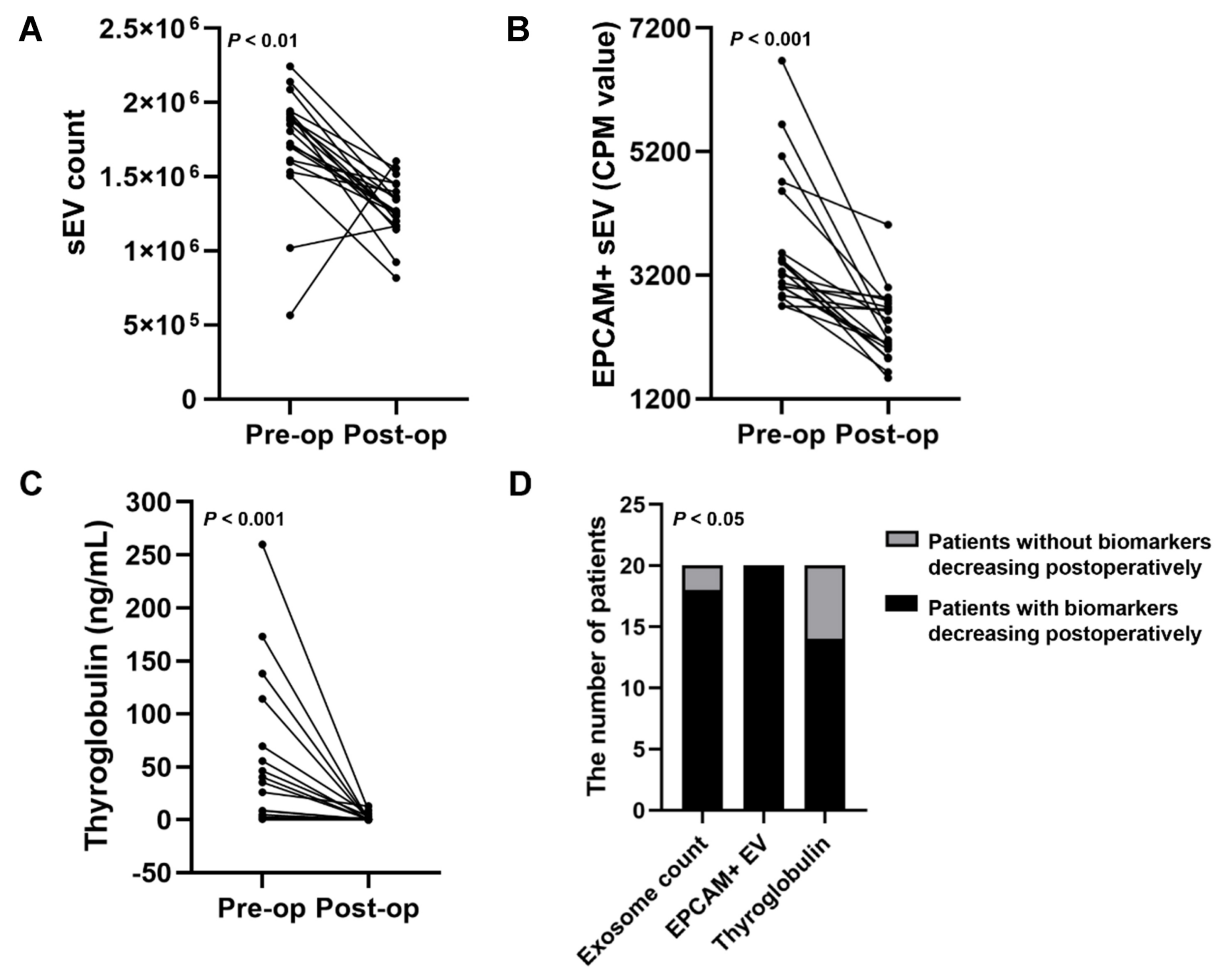
The performance of three blood biomarkers in monitoring post-operative remission of TC. Blood samples of TC patients before and after the operation were collected. The performance of plasma sEV counts (A) and EPCAM^+^ sEVs (B), and serum Tg concentrations (C) in monitoring post-operative remission of TC was analyzed; (D) The chi-square analysis was conducted to evaluate the difference among the three biomarkers in monitoring TC remission. TC: Thyroid carcinoma; sEV: small extracellular vesicle; EPCAM: epithelial cell adhesion molecule; Tg: thyroglobulin; CPM: counts per million.

To investigate the capability of these biomarkers to monitor TC remission, plasma samples from 20 patients with TC before and after the operation were collected for PBA analysis. The 20 patients did not experience TC relapse. The decrease in plasma sEV number was observed in 18 out of 20 patients after surgery [Figure 6A]. Analysis of sEV subpopulations revealed the rapid decline in EPCAM^+^ sEVs in 20 out of 20 patients after the operation [Figure 6B]. The value of the conventional marker, serum Tg, to monitor TC was also determined, which revealed a decrease in serum Tg levels in 14 patients post-operatively [Figure 6C]. The Tg concentrations in preoperative serum samples from 6 of the 20 patients were very low, which may be attributed to the presence of TgAbs. This resulted in the absence of a rapid post-operative decline in Tg levels in these patients. The chi-square analysis showed that the differences among EPCAM^+^ sEVs, sEV counts, and Tg were significant (*P* < 0.05, Figure 6D). These results suggest that EPCAM^+^ sEVs may be superior to serum Tg levels in monitoring the post-operative remission of TC.

### Plasma sEVs from HC suppressed TC cell proliferation, migration, and invasion

**Figure 7:**
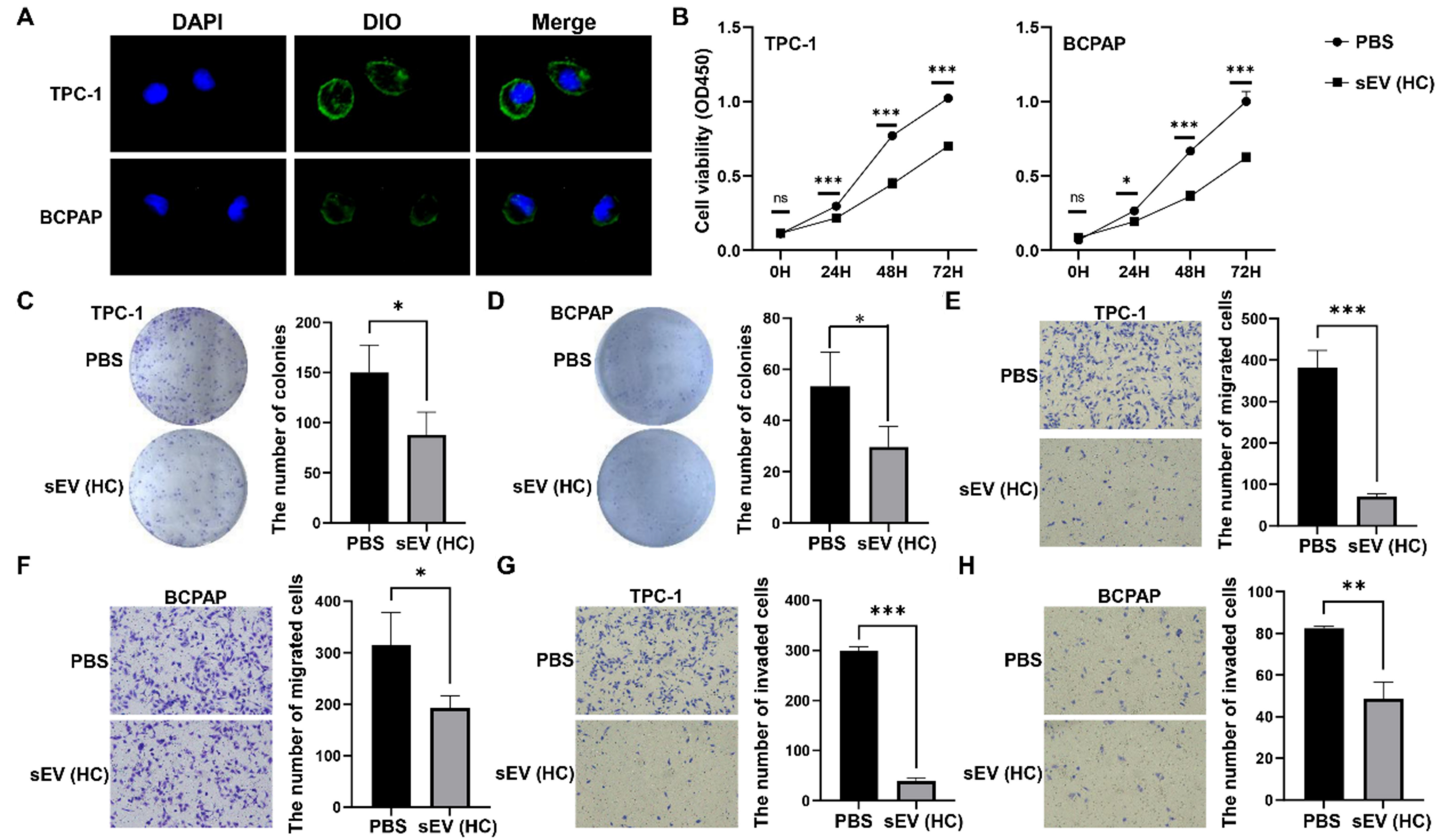
The function of HC-derived plasma sEVs *in vitro*. (A) Dio-labeled plasma sEVs from HC were taken up by TC cells whose nuclei were stained using DAPI; (B) CCK-8 analysis of TC cells treated with PBS or HC-derived plasma sEVs; Representative images of colony formation assays (C and D), migration assays (E and F), and invasion assays (G and H) of TC cells exposed to PBS or HC-derived plasma sEVs. The data was analyzed using the Student’s *t*-test. ^*^P value < 0.05; ^**^P value < 0.01; ^***^P value < 0.001; ns: not significant. HC: Healthy controls; sEVs: small extracellular vesicles; TC: thyroid carcinoma; DAPI: 4′,6-diamidino-2-phenylindole; CCK-8: cell counting kit-8; PBS: phosphate-buffered saline; DIO: 3,3′-dioctadecyloxacarbocyanine perchlorate; OD450: optical density at 450 nm.

We wondered whether plasma sEVs from HC or TC could affect TC cell proliferation, migration, and invasion. The co-localization of DIO-labeled sEVs and DAPI-stained TPC-1 or BCPAP cells showed that the isolated plasma sEVs could be effectively taken up by TPC-1 or BCPAP cells [Figure 7A]. Treatment with plasma sEVs from HC inhibited the proliferation [Figure 7B-D], migration [Figure 7E and F], and invasion [Figure 7G and H] of TPC-1 and BCPAP cells.

### Plasma sEVs from TC promoted TC development *in vitro* and *in vivo*

**Figure 8:**
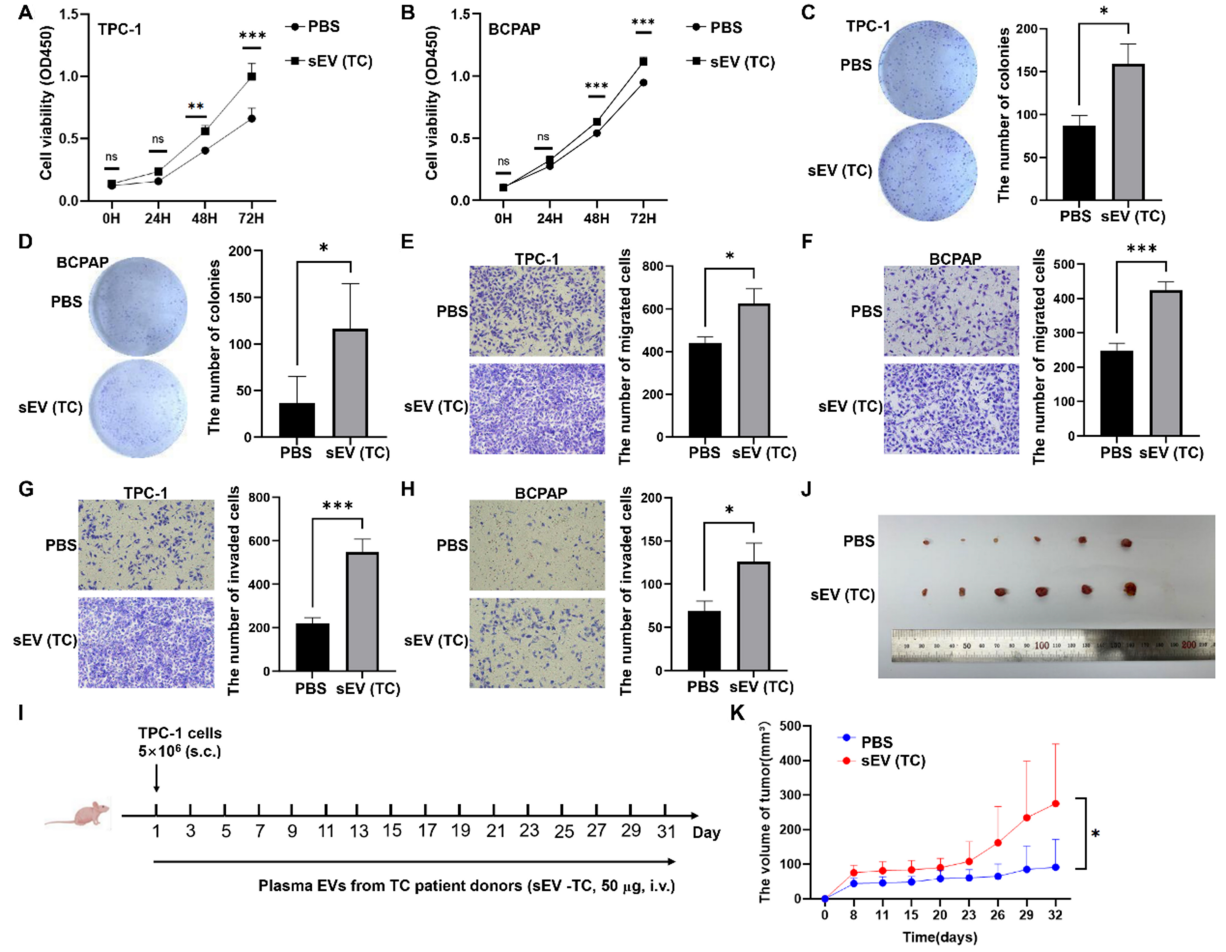
The impact of TC-derived sEVs on TC development *in vitro* and *in vivo*. The effects of TC-derived sEVs on TC cell proliferation determined by CCK-8 assays (A and B) or colony formation assays (C and D); Representative images of migration assays (E and F) and invasion assays (G and H) of TC cells subjected to PBS or plasma sEVs from TC patients; (I) Schematic diagram of mouse experiments; (J) TPC-1 tumors were photographed when the tumor-bearing mice were sacrificed; (K) The tumor volume was compared between two groups. The data was analyzed using the Student’s *t*-test. ^*^P value < 0.05; ^**^P value < 0.01; ^***^P value < 0.001; ns: not significant. TC: Thyroid carcinoma; sEVs: small extracellular vesicles; CCK-8: cell counting kit-8; PBS: phosphate-buffered saline; OD450: optical density at 450 nm.

The functions of TC patient-derived plasma sEVs were analyzed. CCK-8, colony formation, and transwell assays showed that plasma sEVs from patients with TC significantly promoted the proliferation [Figure 8A-D], migration [Figure 8E and F], and invasion [Figure 8G and H] of TPC-1 and BCPAP cells. To determine the effect of TC-derived sEVs on TC tumor growth *in vivo*, TPC-1 cells (5 × 10^6^) were subcutaneously injected into the left axilla of BALB/c nude mice. PBS or TC-derived plasma sEVs were injected into the tail veins of the mice every other day until the mice were sacrificed [Figure 8I]. Plasma sEVs from patients with TC promoted TC xenograft growth compared to that in the control group [Figure 8J and K].

### The function of EPCAM^+^ sEV

**Figure 9:**
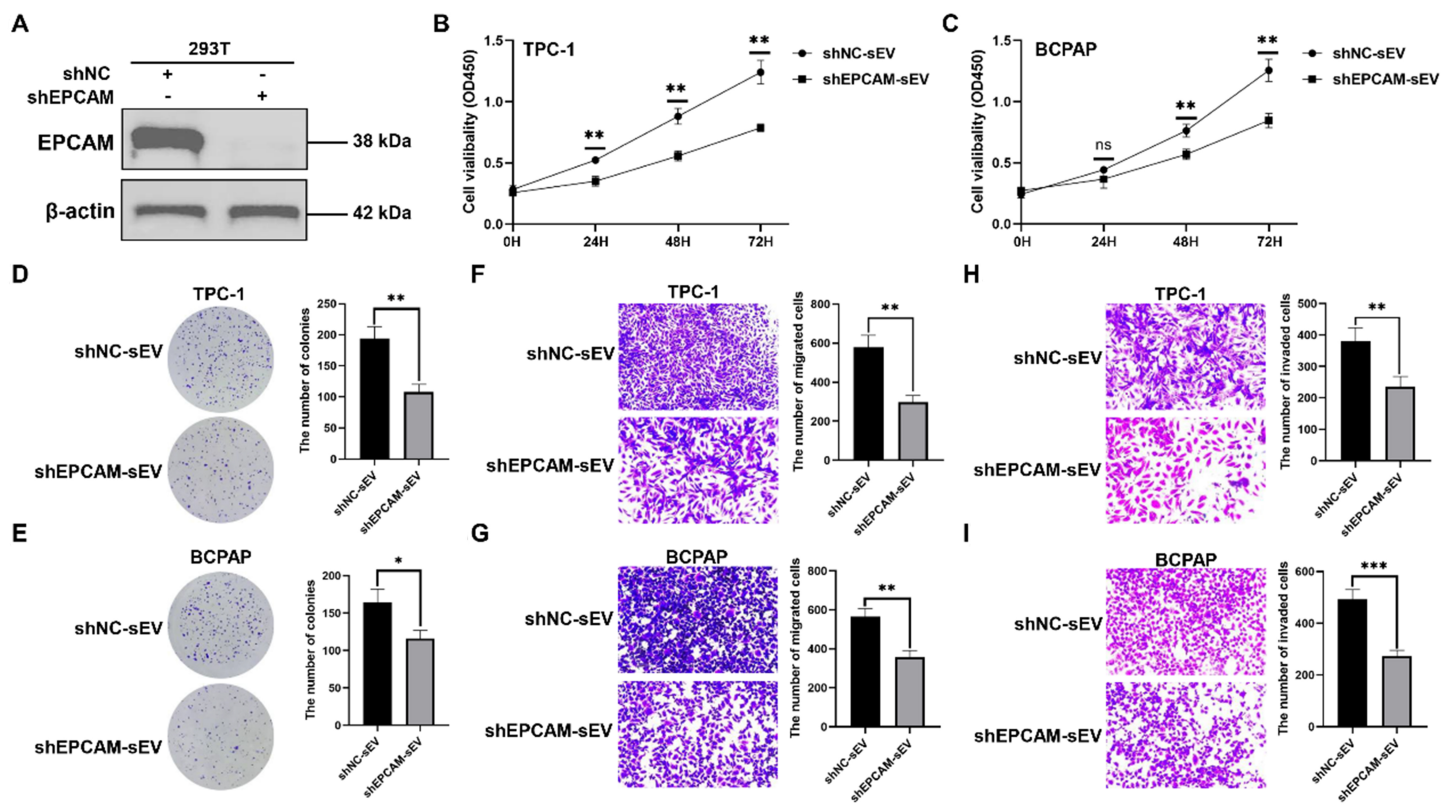
The function of EPCAM^+^ sEV. (A) The western blotting analysis of EPCAM in 293T cells stably transfected with shNC or shEPCAM. The sEVs were isolated from 293T cells stably transfected with shNC or shEPCAM, namely shNC-sEV or shEPCAM-sEV, respectively. TPC-1 and BCPAP cells were treated with shNC-sEV or shEPCAM-sEV; (B and C) TC cell proliferation determined by CCK-8 assays; (D and E) TC cell proliferation determined by colony formation assays; Representative images of migration assays (F and G) and invasion assays (H and I) of TC cells subjected to shNC-sEV or shEPCAM-sEV. The data was analyzed using the Student’s *t*-test. ^*^P value < 0.05; ^**^P value < 0.01; ^***^P value < 0.001; ns: not significant. EPCAM: Epithelial cell adhesion molecule; sEVs: small extracellular vesicles; shNC: lentivirus of negative control short hairpin RNA; shEPCAM: lentivirus of EPCAM short hairpin RNA; TC: thyroid carcinoma; CCK-8: cell counting kit-8; OD450: optical density at 450 nm.

To establish the function of EPCAM^+^ sEV, the 293T cells were stably transfected with shNC or shEPCAM [Figure 9A]. We isolated sEVs from 293T cells stably transfected with shNC or shEPCAM, namely shNC-sEV or shEPCAM-sEV, respectively. TPC-1 and BCPAP cells were treated with these sEVs, and shEPCAM-sEV significantly inhibited TC cell proliferation [Figure 9B-E], migration [Figure 9F and G], and invasion [Figure 9H and I].

## DISCUSSION

Because bulk-level analysis of sEVs could not provide valuable information on sEV heterogeneity, we performed PBA to screen the surface protein profiles of patients with TC at the single-sEV level. We found that the number of sEVs increased in patients with TC, suggesting that enhanced circulating sEV levels may reflect the presence of TC. ROC curve analysis showed that plasma sEV number could function as a robust biomarker for the early screening of TC. It has been reported that tumor cells release elevated amounts of sEVs in patients with glioblastoma^[25]^, melanoma^[26]^, and breast cancer^[27]^. Ricklefs *et al.* reported that the levels of circulating EVs in patients with glioblastoma were elevated 5.5-fold compared to those in healthy donors, and patients with higher levels of circulating EV had significantly shorter progression-free survival than those with lower EV levels^[7]^. They found that EV concentrations decreased rapidly after surgery and that the magnitude of the post-operative drop was related to the extent of tumor resection. Two studies have also reported a notable decline in EV levels in post-operative plasma samples from patients with glioblastoma^[25]^ and head and neck cancer^[28]^. We found that the decrease in sEV counts was observed in 18 out of 20 patients with TC, which surpassed the performance of the conventional marker serum Tg in the surveillance of TC.

EPCAM, also known as CD326, is overexpressed in many human cancers, and its expression is closely associated with EMT of tumor cells^[29,30]^. Due to the epithelial source of EPCAM, EPCAM^+^ sEVs have been demonstrated to be more specifically secreted by epithelial tumors, such as pancreatic ductal adenocarcinoma and ovarian cancer^[31,32]^. TC is a malignancy originating from the epithelium of the thyroid gland. The single-cell transcriptome revealed that thyroid gland contains a large group of EPCAM^+^ epithelial cells, which were identified as thyroid follicular cells, responsible for the synthesis and secretion of thyroid hormones^[33]^. Circulating tumor cells expressing EPCAM are potent biomarkers for distinguishing patients with TC who achieved remission after surgery from those who did not^[34]^. However, whether EPCAM^+^ sEV can be specifically secreted by tumor cells of TC and serve as a biomarker for TC remains uncovered. Our results showed that EPCAM expression was significantly higher in patients with TC than in HC, and sEV EPCAM presented the AUCs > 0.800 in both cohorts. EPCAM^+^ sEV levels were significantly elevated in the TC group. ROC curve analysis showed that EPCAM^+^ sEVs exhibited AUCs > 0.800, suggesting that EPCAM^+^ sEVs could serve as a reliable biomarker for the early diagnosis of TC. Additionally, we found that EPCAM^+^ sEVs were reduced in the post-operative plasma samples from 20 out of 20 patients with TC, indicating that EPCAM^+^ sEVs had a higher accuracy in monitoring post-operative remission of TC compared to that of serum Tg and were also superior to the performance of sEV counts. Thus, the novelty of this study is that we identify EPCAM^+^ sEV as a potential biomarker for discrimination between TC and HC and a promising biomarker for monitoring post-operative remission of TC using PBA.

ITGAX, namely CD11c, is a marker of DCs that function as antigen-presenting cells and activate T cells^[35,36]^. Gu *et al.* reported that tumor sEVs were efficiently taken up by DCs, and that the uptake of tumor sEVs supported upregulation of CD11c, Interleukin 12, and major histocompatibility complex II in DCs^[37]^. CD11c is also critical for the regulation of neutrophil maturation^[35]^. We found that sEV ITGAX expression was significantly lower in patients with TC than in HC, and ITGAX possessed AUCs > 0.800 in both cohorts. These results suggest that sEV ITGAX could serve as a promising biomarker for distinguishing TC from HC. LAG3 is mainly expressed on activated natural killer cells and T cells and mediates the exhaustion of T cells. It became the third inhibitory receptor in the clinic, attracting considerable attention and interest^[22]^. A significant decrease in sEV LAG3 expression was also observed in patients with TC, and LAG3 exhibited AUCs > 0.800 in both cohorts, indicating that sEV LAG3 could function as a potent biomarker to discriminate between TC and HC. Given the expression features of these two proteins, sEVs expressing ITGAX and LAG3 may be mainly derived from DCs and T cells [or natural killer (NK) cells], respectively. The sEVs derived from DCs inherit the antigen presentation ability of their source cells and can activate NK cells, stimulating antitumor immune responses^[38,39]^. The sEVs released by CD8^+^ T cells suppress tumor metastasis and invasion^[38]^, whereas sEVs secreted by NK cells display antitumor activity against several cancers^[40]^.

In this study, we found that plasma sEVs from HC inhibited, whereas sEVs from patients with TC promoted, cell proliferation, migration, and invasion. These observations were in line with the study by Guo *et al.*, who found that the malignancy of CRC cell lines was suppressed by plasma sEVs from healthy donors, but promoted by those from patients with CRC who had metastases in the liver^[14]^. The results of our study and those of Guo *et al.* clearly demonstrate that plasma sEVs possess the features of donors and could influence recipients in a donor-dependent manner.

This study has a few limitations. First, the sample size for PBA was small, resulting in restrictions in broader applicability. We may validate the observations of this study with a larger sample size in the ongoing study. Second, we found that shEPCAM-sEV significantly inhibited TC cell proliferation, migration, and invasion; however, the detailed mechanism by which EPCAM^+^ sEVs promote TC cell proliferation, migration, and invasion remains unclear. Third, this study did not explore the function of EPCAM^+^ sEVs *in vivo*.

In summary, we used the PBA method to analyze the surface protein profiles of patients with TC at the single-sEV level and found that the plasma sEV count served as a noninvasive biomarker for distinguishing TC from HC. Plasma EPCAM^+^ sEVs are a potentially powerful biomarker for the early diagnosis of TC and even surpassed the performance of serum Tg in the post-operative surveillance of TC. The current study was limited by the small sample size. Thus, further investigations in large cohorts are needed. The functions of plasma EPCAM^+^ sEVs are worthy of further research as they may reveal a novel TC therapy.

## DECLARATIONS

### Availability of data and materials

Data will be made available on reasonable request from the corresponding author.

### Copyright

© The Author(s) 2025.

## References

[1] Bray F, Laversanne M, Sung H (2024). Global cancer statistics 2022: GLOBOCAN estimates of incidence and mortality worldwide for 36 cancers in 185 countries. CA Cancer J Clin.

[2] Pellegriti G, Frasca F, Regalbuto C, Squatrito S, Vigneri R (2013). Worldwide increasing incidence of thyroid cancer: update on epidemiology and risk factors. J Cancer Epidemiol.

[3] Allin DM, Shaikh R, Carter P (2018). Circulating tumour DNA is a potential biomarker for disease progression and response to targeted therapy in advanced thyroid cancer. Eur J Cancer.

[4] Spencer CA, Takeuchi M, Kazarosyan M (1998). Serum thyroglobulin autoantibodies: prevalence, influence on serum thyroglobulin measurement, and prognostic significance in patients with differentiated thyroid carcinoma. J Clin Endocrinol Metab.

[5] Paskeh MDA, Entezari M, Mirzaei S (2022). Emerging role of exosomes in cancer progression and tumor microenvironment remodeling. J Hematol Oncol.

[6] Rackles E, Lopez PH, Falcon-Perez JM (2022). Extracellular vesicles as source for the identification of minimally invasive molecular signatures in glioblastoma. Semin Cancer Biol.

[7] Ricklefs FL, Wollmann K, Salviano-Silva A (2024). Circulating extracellular vesicles as biomarker for diagnosis, prognosis, and monitoring in glioblastoma patients. Neuro Oncol.

[8] Li G, Chen W, Jiang K (2024). Exosome-mediated delivery of miR-519e-5p promotes malignant tumor phenotype and CD8+ T-cell exhaustion in metastatic PTC. J Clin Endocrinol Metab.

[9] Zhang H, Freitas D, Kim HS (2018). Identification of distinct nanoparticles and subsets of extracellular vesicles by asymmetric flow field-flow fractionation. Nat Cell Biol.

[10] Higginbotham JN, Zhang Q, Jeppesen DK (2016). Identification and characterization of EGF receptor in individual exosomes by fluorescence-activated vesicle sorting. J Extracell Vesicles.

[11] Lee K, Shao H, Weissleder R, Lee H (2015). Acoustic purification of extracellular microvesicles. ACS Nano.

[12] Bordanaba-Florit G, Royo F, Kruglik SG, Falcón-Pérez JM (2021). Using single-vesicle technologies to unravel the heterogeneity of extracellular vesicles. Nat Protoc.

[13] Wu D, Yan J, Shen X (2019). Profiling surface proteins on individual exosomes using a proximity barcoding assay. Nat Commun.

[14] Guo W, Cai Y, Liu X (2023). Single-exosome profiling identifies ITGB3+ and ITGAM+ exosome subpopulations as promising early diagnostic biomarkers and therapeutic targets for colorectal cancer. Research.

[15] Cai Y, Chen T, Cai Y (2024). Surface protein profiling and subtyping of extracellular vesicles in body fluids reveals non-CSF biomarkers of Alzheimer’s disease. J Extracell Vesicles.

[16] Ning N, Lu J, Li Q (2024). Single-sEV profiling identifies the TACSTD2 + sEV subpopulation as a factor of tumor susceptibility in the elderly. J Nanobiotechnology.

[17] Li N, Tang TT, Gu M (2025). Single urinary extracellular vesicle proteomics identifies complement receptor CD35 as a biomarker for sepsis-associated acute kidney injury. Nat Commun.

[18] Van Gassen S, Callebaut B, Van Helden MJ (2015). FlowSOM: using self-organizing maps for visualization and interpretation of cytometry data. Cytometry A.

[19] Cieslak MC, Castelfranco AM, Roncalli V, Lenz PH, Hartline DK (2020). t-Distributed stochastic neighbor embedding (t-SNE): a tool for eco-physiological transcriptomic analysis. Mar Genomics.

[20] Tian Y, Gong M, Hu Y (2020). Quality and efficiency assessment of six extracellular vesicle isolation methods by nano-flow cytometry. J Extracell Vesicles.

[21] Hashimoto I, Oshima T (2022). Claudins and gastric cancer: an overview. Cancers.

[22] Li CF, Chen JY, Ho YH (2019). Snail-induced claudin-11 prompts collective migration for tumour progression. Nat Cell Biol.

[23] Li D, Guo X, Yang K (2023). EpCAM-targeting CAR-T cell immunotherapy is safe and efficacious for epithelial tumors. Sci Adv.

[24] Andrews LP, Marciscano AE, Drake CG, Vignali DA (2017). LAG3 (CD223) as a cancer immunotherapy target. Immunol Rev.

[25] Osti D, Del Bene M, Rappa G (2019). Clinical significance of extracellular vesicles in plasma from glioblastoma patients. Clin Cancer Res.

[26] Alegre E, Zubiri L, Perez-Gracia JL (2016). Circulating melanoma exosomes as diagnostic and prognosis biomarkers. Clin Chim Acta.

[27] König L, Kasimir-Bauer S, Bittner AK (2017). Elevated levels of extracellular vesicles are associated with therapy failure and disease progression in breast cancer patients undergoing neoadjuvant chemotherapy. Oncoimmunology.

[28] (2019). Zorrilla S, Pérez-Sayans M, Fais S, Logozzi M, Gallas Torreira M, García García A. A pilot clinical study on the prognostic relevance of plasmatic exosomes levels in oral squamous cell carcinoma patients. Cancers.

[29] Sankpal NV, Fleming TP, Sharma PK, Wiedner HJ, Gillanders WE (2017). A double-negative feedback loop between EpCAM and ERK contributes to the regulation of epithelial-mesenchymal transition in cancer. Oncogene.

[30] Ye X, Tam WL, Shibue T (2015). Distinct EMT programs control normal mammary stem cells and tumour-initiating cells. Nature.

[31] Castillo J, Bernard V, San Lucas FA (2018). Surfaceome profiling enables isolation of cancer-specific exosomal cargo in liquid biopsies from pancreatic cancer patients. Ann Oncol.

[32] Zhao Z, Yang Y, Zeng Y, He M (2016). A microfluidic ExoSearch chip for multiplexed exosome detection towards blood-based ovarian cancer diagnosis. Lab Chip.

[33] Liang J, Qian J, Yang L (2022). Modeling human thyroid development by fetal tissue-derived organoid culture. Adv Sci.

[34] Lin JD, Liou MJ, Hsu HL (2018). Circulating epithelial cell characterization and correlation with remission and survival in patients with thyroid cancer. Thyroid.

[35] Hou L, Voit RA, Shibamura-Fujiogi M (2023). CD11c regulates neutrophil maturation. Blood Adv.

[36] Wolff CM, Singer D, Schmidt A, Bekeschus S (2023). Immune and inflammatory responses of human macrophages, dendritic cells, and T-cells in presence of micro- and nanoplastic of different types and sizes. J Hazard Mater.

[37] Gu X, Erb U, Büchler MW, Zöller M (2015). Improved vaccine efficacy of tumor exosome compared to tumor lysate loaded dendritic cells in mice. Int J Cancer.

[38] Li S, Li W, Wu X, Zhang B, Liu L, Yin L (2024). Immune cell-derived extracellular vesicles for precision therapy of inflammatory-related diseases. J Control Release.

[39] Munich S, Sobo-Vujanovic A, Buchser WJ, Beer-Stolz D, Vujanovic NL (2012). Dendritic cell exosomes directly kill tumor cells and activate natural killer cells via TNF superfamily ligands. Oncoimmunology.

[40] Zhu L, Kalimuthu S, Gangadaran P (2017). Exosomes derived from natural killer cells exert therapeutic effect in melanoma. Theranostics.

