# Supplementary material for: A single-sEV analysis identifies plasma EPCAM+ sEVs as a biomarker for early diagnosis and monitoring postoperative remission of thyroid cancer
Source: Extracell Vesicles Circ Nucl Acids. 2025 Dec 15;6(4):982–99. doi: 10.20517/evcna.2025.93 (PMC12809679; doi:10.20517/evcna.2025.93)
Supplement: Supplementary file 1 [file evcna-6-4-982-SupplementaryMaterials.pdf]

Supplementary Materials

A single-sEV analysis identifies plasma EPCAM$^+$ sEVs as a biomarker for early diagnosis and monitoring postoperative remission of thyroid cancer

Simin Yu[1,#], Yuting Luo[2,#], Tianfeng Dang[1,#], Congli Peng[1,#], Qing Gan[1], Yuxuan Liang[1], Jieqing Yu[1], Ping Long[1], Wensheng Zhou[1], Daofeng Dai[1]

[1]Jiangxi Otorhinolaryngology-Head and Neck Surgery Institute, Department of Otorhinolaryngology-Head and Neck Surgery, The First Affiliated Hospital, Jiangxi Medical College, Nanchang University, Nanchang 330006, Jiangxi, China.
[2]Department of General Thyroid Surgery, The First Affiliated Hospital, Jiangxi Medical College, Nanchang University, Nanchang 330006, Jiangxi, China.
[#]Authors contributed equally to this work.

Correspondence to: Dr. Daofeng Dai, Prof. Wensheng Zhou, Prof. Ping Long, Prof. Jieqing Yu, Jiangxi Otorhinolaryngology-Head and Neck Surgery Institute, Department of Otorhinolaryngology-Head and Neck Surgery, The First Affiliated Hospital, Jiangxi Medical College, Nanchang University, Nanchang 330006, Jiangxi, China. E-mail: daofengdai@ncu.edu.cn; zhouwesh@163.com; yifuyuanlongping@163.com; yujieq@ncu.edu.cn

Supplementary Table 1. The clinicopathological features of TC patients recruited in this study

Variable	Cohort 1	Cohort 2	Cohort 3	Cohort 4
**Sex**				
Male	3	6	6	4
Female	7	22	28	17
**Age (years, mean ± SD)**	44.50 ± 7.56	39.25 ± 12.62	47.32 ± 9.46	45.62 ± 11.91
**Histological subtype**				
**PTC**	10	28	34	21
**Size (cm)**				
< 1	2	2	26	12
≥ 1	8	26	8	9
**T stage**				
T1	5	21	33	17
T2	4	6	0	3
T3	0	1	0	0
T4	1	0	1	1
**N stage**				
N0	6	10	22	10
N1-N2	4	18	12	11
**Clinical stage**				
I	9	28	34	21
II	0	0	0	0
III	1	0	0	0
**Extrathyroidal extension**				
Yes	1	1	1	9
No	9	17	33	12

TC, thyroid cancer; PTC, papillary thyroid cancer.

Supplementary Table 2. The top 20 significantly differentially expressed proteins in Cohort 1

Protein	UniProt ID
CD151	P48509
CD26	P27487
CEACAM8	P31997
EPCAM	P16422
MADCAM1	Q13477
NLGN1	Q8N2Q7
SELE	P16581
Thy1	P04216
CAV1	Q03135
CDH2	P19022
CDON	Q4KMG0
CLDN10	P78369
CLDN11	O75508
CLEC5A	Q9NY25
GPA33	Q99795
GPC1	P35052
ITGAX	P20702
LAG3	P18627
PROM1	O43490
TPBG	Q13641

Supplementary Table 3. The top 20 significantly differentially expressed proteins in Cohort 2

Protein	UniProt ID
CD44	P16070
CEACAM5	P06731
CEACAM7	Q14002
CPM	P14384
EPCAM	P16422
ITGB3	P05106
NT5E	P21589
PCDH1	Q08174
SIGLEC6	O43699
TENM1	Q9UKZ4
ALDH1A1	P00352
CDH13	P55290
CDH6	P55285
CLDN11	O75508
EMCN	Q9ULC0
FN1	P02751
IL1RAPL1	Q9NZN1
ILK	Q13418
ITGAX	P20702
LAG3	P18627

Supplementary Table 4. The top 20 significantly differentially expressed sEV in Cohort 1

sEV	UniProt ID
$CD26^+$ sEV	P27487
$EPCAM^+$ sEV	P16422
$SIGLEC11^+$ sEV	Q96RL6
$PCDH17^+$ sEV	O14917
$CEACAM8^+$ sEV	P31997
$CDON^+$ sEV	Q4KMG0
$CLDN10^+$ sEV	P78369
$LAG3^+$ sEV	P18627

Supplementary Table 5. The top 20 significantly differentially expressed sEV in Cohort 2

sEV	UniProt ID
TENM2$^+$ sEV	Q9NT68
EPCAM$^+$ sEV	P16422
ESAM$^+$ sEV	Q96AP7
FN1$^+$ sEV	P02751
CDH13$^+$ sEV	P55290
SIGLEC11$^+$ sEV	Q96RL6
MUC1$^+$ sEV	P15941
MMP9$^+$ sEV	P14780
CDH1$^+$ sEV	P12830
TENM4$^+$ sEV	Q6N022
LAG3$^+$ sEV	P18627
EMCN$^+$ sEV	Q9ULC0
Thy1$^+$ sEV	P04216

Supplementary Table 6. Featured proteins in exosome clusters in Cohort 1

Cluster	Protein
Cluster 1	TENM2
Cluster 2	ESAM
Cluster 3	CDH13
Cluster 4	CD36
Cluster 5	SIGLEC9
Cluster 6	ITGB4
Cluster 7	MUC1
Cluster 8	PCDH17
Cluster 9	EPCAM
Cluster 10	CLDN4
Cluster 11	CD36, ITGA1
Cluster 12	ULBP1
Cluster 13	SIGLEC11
Cluster 14	NECTIN1
Cluster 15	LICAM
Cluster 16	DSC1
Cluster 17	ITGA1
Cluster 18	FN1

Supplementary Table 7. Featured proteins in exosome clusters in Cohort 2

Cluster	Protein
Cluster 1	ITGA1, ITGAV
Cluster 2	DSC1, ITGB4
Cluster 3	DSC1, FN1
Cluster 4	ITGA1, DSC2
Cluster 5	ITGB4
Cluster 6	DSC1
Cluster 7	MUC1
Cluster 8	ADAM10
Cluster 9	DSGI
Cluster 10	FOLH1
Cluster 11	TENM2
Cluster 12	LICAM
Cluster 13	ESAM
Cluster 14	ULBP1
Cluster 15	EPCAM
Cluster 16	CLDN4
Cluster 17	SIGLEC9
Cluster 18	PCDH17
Cluster 19	CDH13
Cluster 20	SIGLEC11
